# Uncovering the Metabolic and Stress Responses of Human Embryonic Stem Cells to *FTH1* Gene Silencing

**DOI:** 10.3390/cells10092431

**Published:** 2021-09-15

**Authors:** Luana Scaramuzzino, Valeria Lucchino, Stefania Scalise, Michela Lo Conte, Clara Zannino, Alessandro Sacco, Flavia Biamonte, Elvira Immacolata Parrotta, Francesco Saverio Costanzo, Giovanni Cuda

**Affiliations:** 1 Research Centre for Advanced Biochemistry and Molecular Biology, Department of Experimental and Clinical Medicine, University Magna Graecia, 88100 Catanzaro, Italy; scaramuzzino.luana@unicz.it (L.S.); valeria.lucchino@unicz.it (V.L.); stefania.scalise@unicz.it (S.S.); michela.loconte@studenti.unicz.it (M.L.C.); zanninoclara@gmail.com (C.Z.); a.sacco@unicz.it (A.S.); flavia.biamonte@unicz.it (F.B.); fsc@unicz.it (F.S.C.); cuda@unicz.it (G.C.); 2 Center of Interdepartmental Services (CIS), “Magna Graecia” University of Catanzaro, 88100 Catanzaro, Italy; 3 Department of Medical and Surgical Sciences, University Magna Graecia, 88100 Catanzaro, Italy

**Keywords:** human embryonic stem cells (hESCs), FTH1, redox regulation, oxidative stress, Nrf2, metabolic rerouting

## Abstract

Embryonic stem cells (ESCs) are pluripotent cells with indefinite self-renewal ability and differentiation properties. To function properly and maintain genomic stability, ESCs need to be endowed with an efficient repair system as well as effective redox homeostasis. In this study, we investigated different aspects involved in ESCs’ response to iron accumulation following stable knockdown of the ferritin heavy chain (FTH1) gene, which encodes for a major iron storage protein with ferroxidase activity. Experimental findings highlight unexpected and, to a certain extent, paradoxical results. If on one hand FTH1 silencing does not correlate with increased ROS production nor with changes in the redox status, strengthening the concept that hESCs are extremely resistant and, to a certain extent, even refractory to intracellular iron imbalance, on the other, the differentiation potential of hESCs seems to be affected and apoptosis is observed. Interestingly, we found that FTH1 silencing is accompanied by a significant activation of the nuclear factor (erythroid-derived-2)-like 2 (Nrf2) signaling pathway and pentose phosphate pathway (PPP), which crosstalk in driving hESCs antioxidant cascade events. These findings shed new light on how hESCs perform under oxidative stress, dissecting the molecular mechanisms through which Nrf2, in combination with PPP, counteracts oxidative injury triggered by FTH1 knockdown.

## 1. Introduction

An impaired intracellular balance between reactive oxygen species (ROS) production and clearance is responsible for establishing an oxidative stress status, eventually leading to lethal consequences [1] for the cells. ROS are able to elicit a wide spectrum of responses; slight variations in their concentration may produce profound and opposite effects in distinct cell types [2]. High levels of ROS, predominantly produced by mitochondrial respiration, are classically linked to cellular damage, DNA damage, and apoptosis. At the same time, a large body of data recognizes ROS as signaling messengers involved in many biological processes, including DNA synthesis, modulation of gene expression, cellular respiration, protein–protein interactions [3], as well as activation of critical pathways such as protein kinase B (AKT) and NF-κB signaling [4]. In hESCs, constant exposure to high levels of ROS was shown to induce cell cycle arrest and apoptosis [5]; however, by regulating the redox state, ROS also target important aspects of pluripotency, such as metabolism, self-renewal, and cell fate [6,7,8]. During evolution, cells have developed specific and efficient antioxidant defense mechanisms to promptly detoxify reactive intermediates and to maintain redox homeostasis [9]. The response to oxidative stress is quite variable: some cell types are extremely sensitive, while others, such as ESCs, are highly resistant [5,10]. ESCs are pluripotent cells characterized by an unlimited capacity for self-renewal and the ability to give rise to cells of all three germ layers (ectoderm, mesoderm, and endoderm) that occur naturally during embryo development in vivo, or by differentiating culture conditions in vitro [11]. As such, it is extremely important for ESCs to function properly and to maintain their genome stability. To do so, ESCs are equipped with enhanced repair systems and efficient control of redox homeostasis to prevent dramatic consequences of oxidative stress [12]. It has been shown that low levels of ROS are required for ESCs to sustain their self-renewal capability [10,13], while a significant increase in ROS promotes ESC differentiation [14,15]. The canonical pluripotency core itself, including *OCT4*, *SOX2*, and *NANOG*, is responsible for regulating the expression of some important antioxidant genes, such as *SOD1*, suggesting a correlation between mechanisms governing pluripotency and antioxidant systems [16]. To maintain high antioxidant capacity, ESCs rely on low mitochondrial biogenesis and reduced oxygen consumption [17,18,19]. The antioxidant system includes a wide range of enzymes such as those encoded by the superoxide dismutase family genes (*SODs*), glutathione peroxidase family (*GPXs*), and catalase (*CAT*). In addition, many other non-enzymatic molecules and compounds such as ascorbic acid, directly or indirectly, participate in antioxidant events [20].

The redox status is greatly influenced by compounds primarily involved in the metabolism of specific substances, such as iron [21]. Iron deficiency, triggered by deferoxamine (DFO) treatment, causes a loss of pluripotency in both ESCs and induced pluripotent stem cells (iPSCs) [22]. On the other hand, increased intracellular iron levels negatively affect the status of pluripotent stem cells (PSCs), leading to excess ROS production and DNA damage and a reduced proliferation rate [23]. The effects of ROS accumulation in ESCs and their functional consequences remain controversial. The role of unbalanced iron metabolism in ESCs is even less well understood, suggesting that much still remains to be investigated in this direction. 

PSCs mainly rely on glycolysis [24], while oxidative metabolism (i.e., mitochondrial respiration and fatty acid metabolism) take place upon PSC differentiation [17,25,26]. Reprogramming of somatic cells to generate iPSCs requires, among others, a metabolic switch from oxidative phosphorylation (OXPHOS) to glycolysis [18]. The prevailing aerobic over oxidative mitochondrial metabolism in PSCs is reminiscent of cancer metabolism [27,28,29]. Like cancer cells, in fact, PSCs divide rapidly and, in order to sustain a high proliferation rate, these cells are required to produce ATP from glycolysis even in the presence of normal oxygen levels. Glycolytic metabolism also generates nicotinamide adenine dinucleotide phosphate (NADPH), which helps to protect cells from oxidative stress injuries [30]. Additionally, as cancer cells, PSCs are characterized by an increased expression of hypoxia-inducible factors (HIFs) and oxygen-sensitive transcription factors. Under particular conditions, such as in the presence of high concentrations of lipids, oxidative mitochondrial metabolism is active in hESCs, indicating that these cells can rapidly adapt their metabolism to various nutrient conditions while maintaining full self-renewal properties [31]. 

To push PSC technology towards clinical applications, a comprehensive understanding of stemness and its potentiality, as well as stem cell metabolism and its modulation, is fundamental. While ESCs’ general metabolic aspects have been widely investigated [31,32,33], very little is currently known about the role of iron metabolism and the consequences of iron accumulation in hESCs. On the basis of these considerations, the present work aims to dissect the molecular events triggered by silencing of the FTH1 gene, encoding a major antioxidant protein with ferroxidase activity. We have analyzed key processes known to be associated with intracellular iron accumulation, such as DNA damage, apoptosis, oxidative stress, antioxidant defense system, and metabolic changes. Our findings provide clear evidence of an overactivation of the nuclear factor (erythroid-derived-2)-like 2 (Nrf2) signaling pathway, which emerges as the major player and safeguard against iron-mediated oxidative stress in response to downregulation of ferritin expression. From a metabolic point of view, we also identified pentose phosphate pathway (PPP) activation as the primary metabolic response to maintain the redox status in hESCs.

## 2. Materials and Methods

### 2.1. Cell Culture

H9 Embryonic Stem Cell line was obtained from WiCell Research Institute (Madison, WI, USA, http://www.wicell.org, accessed on 30 April 2019). ESCs were propagated on Matrigel-coated (Corning, New York, NY, USA, 354277) dishes in StemMACS iPS-Brew medium (Miltenyi Biotec, Bergisch Gladbach, Germany, 130-104-368) and cultured at 37 °C in 5% CO_2_ in a humidified incubator with daily medium replacement. Cells were sub-cultured as small colonies with Gentle Dissociation reagent (STEMCELL Technologies, Vancouver, BC, Canada, 07174).

### 2.2. Lentiviral Production and Generation of Stable Knock-Down ESCs

Lentiviral particles were produced in 293T cells following the standard procedure. Briefly, sub-confluent 293T cells were transfected with shFTH1 or shSCR control lentiviral vector plasmids (Sigma-Aldrich, St. Louis, MO, USA) along with pCMV-DR9-9 and pCMV-VSVG plasmids (Sigma-Aldrich). After 48 h, supernatants were harvested, filtered through a 0.45 µM filter, and used for ESCs infection, previously seeded in a 6-well plate at a cell density of 2 × 105 cells/well. Infected cells were incubated with shFTH1 and shSCR control lentiviral particles [Multiplicity of Infection (MOI) = 10] in the presence of 8 μg/mL of Polybrene (Sigma-Aldrich) using Lipofectamine 3000 Reagent (Thermo Fisher Scientific, Waltham, MA, USA). Two days after transduction, transduced cells were selected with puromycin (1 µg/mL) for 10 days. Knock-down efficiency was assessed via both Western blot and qRT-PCR analysis (Figure S1).

### 2.3. RNA Isolation and Quantitative Real-Time PCR

Cell pellets were collected after 48 h of culture and total RNA was extracted with TRIzol Reagent (Thermo Fisher Scientific, 15596018) and reverse transcribed using High-Capacity cDNA Reverse Transcription kit (Applied Biosystems, Foster City, CA, USA, 4368814) according to manufacturer’s instructions. Real-time quantitative PCR (qRT-PCR) was performed according to StepOnePlus system’s protocol (Applied Biosystems) with SensiFAST SYBR Hi-ROX Kit (Meridian Bioscience, Cincinnati, OH, USA, BIO-92020). Ct values for each target gene were normalized to GAPDH Ct values. See Supplement Information Table S1 for the primer sequences used in this study.

### 2.4. Immunofluorescence

Cells at ~80% confluence were fixed with 3.7% formaldehyde (Sigma-Aldrich), permeabilized with 0.1% Triton X-100, then blocked with 1% bovine serum albumin (BSA) in PBS. Cells were stained with primary antibodies Nanog (1:200; rabbit polyclonal, #PA1-097, Thermo Fisher Scientific), Oct4 (1:200; mouse monoclonal, #75463, Cell Signalling), Sox2 (1:500; rabbit monoclonal, #97959, Abcam), TRA1-60 (1:100; mouse monoclonal, #41-100, Life Technologies, Carlsbad, CA, USA), Brachyury (1:20; goat polyclonal, #AF2085, R&D System), Sox17 (1:20; goat polyclonal, #AF1924, R&D System), Nestin (1:1000; mouse monoclonal, #60091, Stem Cell Technologies), and Nrf2 (1:100; mouse monoclonal, #SC-365949, Santa Cruz) and incubated overnight at 4 °C in a blocking buffer. Secondary antibodies goat anti-mouse Alexa-Fluor-488, goat anti-rabbit Alexa Fluor-594, and donkey anti-goat Alexa-Fluor-594 (all from Life Technologies) were used for detection. DAPI (Carl Roth) was used for nuclei counterstain. Images were acquired with a Leica DMi8 inverted microscope. The filter cubes and software were provided by Leica. 

### 2.5. Western Blotting

Cells were harvested after reaching 90% confluency and then were lysed in RIPA lysis buffer (Sigma-Aldrich) containing a protease inhibitor cocktail (Thermo Fisher Scientific, 78430). Protein concentration was determined by Bradford assay (Biorad, Hercules, Canada). Equal amounts of protein were resolved by 12% SDS-PAGE and transferred to a nitrocellulose membrane (Biorad, 1704159). Membranes were blocked at room temperature with TBST (0.1% Tween-20 in TBST) containing 5% milk. Immunoblotting was performed with primary antibodies against FTH1 (Santa Cruz, #376594, 1:200), Nanog (Thermo Fisher Scientific, #PA1-097, 1:500), Nrf2 (Santa Cruz, #SC-365949, 1:100), p62/Sqstm1 (Abcam, #ab56416, 1:1000), Gpx4 (Abcam, #ab41787, 1 µg/mL), HIF1α (Cell Signaling, #3716s, 1:1000), FTL (Santa Cruz, #74513, 1:100), Caspase 3 (Cell Signaling, #9669, 1:1000), Akt1 (Cell Signaling, #2967, 1:1000), pAkt1 (Cell Signaling, #4058, 1:1000), Erk1/2 (Cell Signaling, #9107, 1:1000), pErk1/2 (Cell Signaling, #4376, 1:1000), and Parp1 (Cell Signaling, #9543, 1:1000) overnight at 4 °C. After several washes with TBST, membranes were incubated with horseradish peroxidase (HRP)-conjugated secondary antibodies (Jackson ImmunoResearch, West Grove, PA, USA) for 1 h at room temperature. Immunoreactive protein bands were probed using an enhanced chemiluminescence detection system (Biorad, Hercules, CA, USA, 170-5060) and acquired with UVITEC 165 Imaging Systems. Gapdh (Bioss, BS-1099 R, 1:1000) or Actin (Santa Cruz, SC-1616, 1:500) antibodies were used indifferently as loading control.

### 2.6. Measurement of Intracellular Iron, Total ROS and Mitochondrial Superoxide Species

Intracellular iron concentration was measured using the Iron Assay kit (Sigma-Aldrich, MAK025) according to the manufacturer’s instructions. Total intracytoplasmic ROS as well as specific mitochondrial superoxide species were assessed by flow cytometry using the fluorescent probes 2′-7′- Dichlorodihydrofluorescein diacetate (CM-H2DCFDA; Thermo Fisher Scientific) and MitoSOX™ Red Mitochondrial Superoxide Indicator, respectively. For the quantification of mitochondrial superoxide levels, cells were cultured for 72 h and then incubated with 2 mM MitoSOX™ Red Mitochondrial Superoxide Indicator (Thermo Fisher Scientific) for 30 min at 37 °C. Intracellular ROS levels were instead assayed by staining the cells with 1.5 mM CM-H2DCFDA (Thermo Fisher Scientific) for 30 min at 37 °C. Fluorescence intensity was measured by flow cytometry using a FACS BD LSRFortessaTM X-20 cytofluorimeter (BD Biosciences, Bedford, MA, USA). The results were analyzed with FlowJo software (Tree Star, Inc., Williamson Way Ashland, OR 97520, USA). Three independent experiments were conducted.

### 2.7. Analysis of Mitochondrial Membrane Potential (*ΔΨ*)

Mitochondrial membrane potential (ΔΨ) was measured using 0.1 µM ΔΨ fluorescent indicator TMRM (tetramethylrhodamine methyl ester) (Thermo Fisher Scientific). Cells were harvested after 72 h of culture and TMRM fluorescence intensity was measured after 30 min of incubation at 37 °C by flow cytometry using a FACS BD LSRFortessaTM X-20 cytofluorimeter (BD Biosciences). The results were analyzed with FlowJo software (Tree Star, Inc.). Three independent experiments were conducted.

### 2.8. Embryoid Bodies (EBs) Formation Assay

ESCs at ~80% confluence were dissociated into single cells with StemPro Accutase (Life Technologies). A total of 1.5 × 10^6^ cells were resuspended in Brew medium containing 10 µM Y27632 ROCK inhibitor (Selleckchem, S1049), and cultured on ultra-low attachment plates (TPP, 93060). Cells were maintained in these conditions for 3 days. After 7 days, floating EBs were plated on laminin-coated dishes (Biolamina, Sundbyberg, Sweden, LN521-03) and cultured in DMEM-F12 medium (Gibco, Waltham, MA, USA, 21331-020) containing 20% Knockout Serum Replacement (KSR; Gibco, 10828-028), 1% Glutamax I-CTS (Gibco, A12860-01), 1% MEM-NEAA (Gibco, 1140-035), 0,1 mM 2-mercaptoethanol (Gibco, 21985-023), and 1% Pen/Strep (Gibco, 15140-122) for 28 days.

### 2.9. Alkaline Phosphatase Assay

After 48 h of culture, cells were fixed with 3.7% formaldehyde (FA) solution (Sigma Aldrich, 252549-100ML) for 15 min at room temperature. Alkaline phosphatase assay was performed using 1-Step NBT/BCIP (Thermo Fisher Scientific, 34042) according to the manufacturer’s instructions.

### 2.10. Metabolic and Bioenergetic Function Profile

Total ATP production rates in H9-shFTH1 and shSCR control cells were measured with the Agilent Seahorse analyzer using the XFp Real-Time Rate Assay Kit. Simultaneous quantification of ATP fractions produced by mitochondrial oxidative phosphorylation (OXPHOS) and glycolysis were detected. For bioenergetics quantification, cells were dissociated into single cells with Accutase and seeded onto Agilent Seahorse XFp Cell Culture Miniplates at a cell density of 2 × 10^3^/well in brew medium supplemented with 10 µM Y27632 ROCK inhibitor. After 24 h, the growth medium was exchanged with unbuffered media and incubated for 30 min at 37 °C, allowing temperature and pH to reach their equilibria before starting the baseline measurements. Once the basal oxygen consumption rate (OCR) and extracellular acidification rate (ECAR) were obtained, cells were metabolically perturbed by the addition of three different compounds: oligomycin (1.5 µM), rotenone, and antimycin A (0.5 µM). Finally, the template was loaded into the Seahorse Analyzer for ATP measurement. The same procedure for cell preparation was used for the glycolytic rate assay and measurement performed according to the manufacturer’s instruction. Rotenone/antimycin A were used to block mitochondrial activity while 2-DG was used to inhibit glycolysis. Results were uploaded to Agilent Seahorse Analytics to calculate parameters relative to ATP production and XF glycolytic rate assays. GraphPad Prism software was used to create graphs. 

### 2.11. Apoptosis Analysis

For the identification of apoptotic cells, a double staining with Annexin V and PI was performed using Alexa Fluor^®^488 Annexin V/Dead Cell Apoptosis Kit (Thermo Fisher Scientific, Waltham, MA, USA) according to the manufacturer’s instructions. 72 h after seeding, cells were harvested and stained with Annexin V and PI, alone and in combination. After an incubation of 15 min at room temperature, each tube was diluted with 400 µL of annexin binding buffer and cells were analyzed by flow cytometry using BD LSRFortessa^TM^ X-20 cytofluorometer (BD Biosciences).

### 2.12. Statistics

All statistical data were generated from at least three independent biological replicates and are represented as mean ± standard error of the mean (SEM). Data were analyzed with PRISM version 7.0 (GraphPad Software Inc., San Diego, CA, USA). The significance of differences (*p*-value) was analyzed using Student’s t-test and presented with the following levels of significance: * *p* < 0.05, ** *p* < 0.01, and *** *p* < 0.001.

## 3. Results

### 3.1. Effects of FTH1-Silencing on ESCs Pluripotency

**Figure 1:**
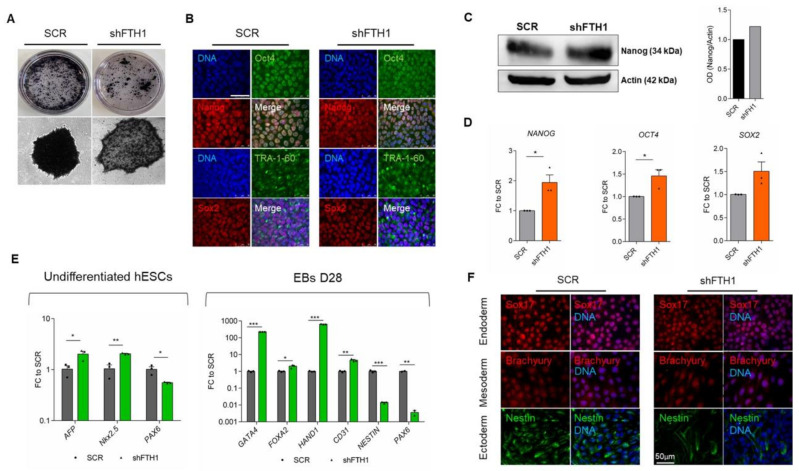
*FTH1* silencing influences hESCs pluripotency. (**A**) Alkaline phosphatase (AP) staining of colonies in SCR control and in hESCs transfected with lentiviral shFTH1 shows that FTH1-silenced ES colonies contain less AP+ cells; scale bar, 250 µm; (**B**) Undifferentiated SCR and FTH1-KD hESCs were stained for Oct4 (green), Nanog (red), TRA-1-60 (green), and Sox2 (red) expression, while nuclei were counterstained with 4′.6-diamidino-2-phenylindole (DAPI). Scale bars, 50 μm. No significant differences were detected between the two groups with the exception of Nanog, which shows a slight positivity in FTH1-silenced cells; (**C**) The levels of Nanog protein were assessed by immunoblotting (IB). Band intensity analysis shows a higher level in FTH1-KD cells compared to SCR control. Actin levels were evaluated to confirm equal loading control; (**D**) mRNA levels of pluripotency-associated genes *NANOG*, *OCT4*, and *SOX2* were measured in undifferentiated SCR and FTH1-KD hESCs via qRT-PCR; (**E**) left panel: mRNA levels of endodermal (*AFP*), mesodermal (*Nkx2.5*), and ectodermal (*PAX6*) genes were measured in undifferentiated SCR and FTH1-silenced hESCs via qRT-PCR analysis; *AFP* and *Nkx2.5* expression levels were higher in shFTH1 cells, while the expression levels of *PAX6* were significantly diminished in silenced cells; (**E**) right panel: mRNA levels of endodermal (*GATA4*, *FOXA2)*, mesodermal (*HAND1*, *CD31*), and ectodermal (*NESTIN*, *PAX6*) genes were measured in SCR and FTH1-KD embryoid bodies (EBs) on day 28 of differentiation by qRT-PCR; (**F**) Sox17, Brachyury, and Nestin, endodermal, mesodermal, and ectodermal markers, respectively, were stained via immunofluorescence in SCR and shFTH EBs on day 28 of differentiation; scale bar, 50 μm. Results of all qRT-PCR shown were normalized to value in SCR cells and are represented as mean ± SEM (*n* = 3); significance is calculated by *t*-test: * *p* ≤ 0.05; ** *p* ≤ 0.01, *** *p* ≤ 0.001.

Iron homeostasis is involved in many biological processes, including maintenance of pluripotency in human PSCs. Results from previous work found that depletion of intracellular iron is associated with impairment of pluripotency and self-renewal due to a significant reduction in *NANOG* expression [22]. Cells maintain a balanced iron pool and ferritin, as the major iron storage protein, plays a crucial role in this regulation. Therefore, to speculate on the role of ferritin in the context of hESCs, we first generated FTH1-silenced cells (FTH1-KD) via lentiviral transfection with short hairpin RNA (shRNA), confirming the successful knock-down both at the mRNA and protein level of the transfected hESC (Supplementary Figure S1B–E). Morphologically, colonies originated from FTH1-KD hESCs were more flattened than those of shSCR control (Supplementary Figure S1A), and alkaline phosphatase (AP) activity was reduced in these cells (Figure 1A). Nevertheless, FTH1-KD hESCs colonies were positive for pluripotency markers Oct4, TRA-1-60, and Sox2, while the expression of Nanog was slightly increased (Figure 1B). This last finding was further confirmed by immunoblot analysis, demonstrating that Nanog expression is indeed higher in FTH1-silenced hESCs. (Figure 1C and Supplementary Figure S2A). At the transcriptional level, *NANOG*, *OCT4*, and *SOX2* resulted in evenly increased FTH1-KD hESCs (Figure 1D). We further asked whether *FTH1* knockdown could, either directly or indirectly, promote spontaneous differentiation of hESCs. Analysis of the transcript levels of *AFP*, *Nkx2*.5, and *PAX6*, specific to endoderm, mesoderm, and ectoderm germ layers, respectively, revealed an enhanced expression of these genes in FTH1-KD hESCs (Figure 1E, left panel). Next, we induced both SCR control and FTH1-KD hESCs to differentiate using the embryoid bodies (EBs) formation assay (Supplementary Figure S2B). On day 28 of differentiation, EBs were harvested and analyzed for endodermal- (*GATA4*, *FOXA2*), mesodermal- (*HAND1*, *CD31*), and ectodermal- (*NESTIN*, *PAX6*) associated markers. Interestingly, while endodermal and mesodermal transcript levels resulted in upregulated FTH1-KD-derived EBs compared to control EBs, the ectodermal genes *NESTIN* and *PAX6* were significantly downregulated in EBs derived from silenced cells (Figure 1E, right panel). Immunofluorescence analysis for Sox17 (endodermal marker), Brachyury (mesodermal marker), and Nestin further confirmed that the percentage of Nestin^+^ cells is lower in FTH1-silenced hESCs compared to SCR control (Figure 1F and Supplementary Figure S2C). We do not have a clear-cut explanation for the contradictory findings reported here; we hypothesize that the initial response of ESCs to FTH1 gene silencing might be overcome during EB formation, in particular towards mesodermal and endodermal differentiation. The molecular mechanisms underlying this controversial phenomenon are still under deep investigation by our group.

### 3.2. ROS Levels Are Reduced upon FTH1-Gene Silencing in hESCs

**Figure 2:**
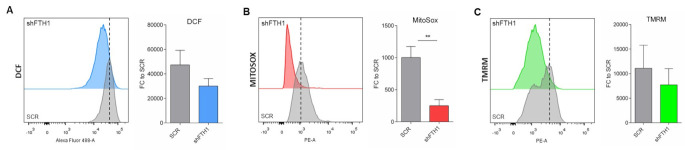
Analysis of ROS levels and mitochondrial functionality in FTH1-KD hESCs. (**A**) DCF staining showed that total cellular ROS levels are reduced in FTH1-silenced hESCs (light blue curve and bar plot) with respect to control cells; (**B**) Mitochondrial superoxide was significantly lower in FTH1-silenced hESCs (red curve and bar plot); (**C**) FTH1-KD cells presented a decrease in mitochondrial membrane depolarization (green curve and bar plot) compared to SCR cells. All data are represented as mean ± SEM (*n* = 3); ** *p* ≤ 0.01, *t*-test. Small dots refer to SCR replicates, while squares refer to shFTH1 triplicates.

The role of ferritin in preventing iron-mediated oxidative stress has been reported in several studies [34]. Downregulation in *FTH1* expression leads to excess labile iron, in turn promoting the formation of oxygen-derived free radicals [35,36]. To speculate on the effects of *FTH1* silencing in hESCs, we measured the levels of intracellular iron in these cells; moreover, we performed a measurement of total and mitochondrial ROS levels together with an analysis of the mitochondrial membrane potential (ΔΨM) on three biological replicates of SCR vs. FTH1-KD hESCs, using DCF, MitoSox, and TMRM dyes, respectively (Supplementary Figure S3B,C). Intriguingly, while iron concentration increased, as expected, in FTH1-KD hESCs (Supplementary Figure S3A), a significant reduction in total ROS levels was detected compared to SCR controls (FTH1-KD: CM-H2DCFDA MFI: 29785,7 vs. SCR: CM-H2DCFDA MFI: 47341,7) (Figure 2A). Likewise, MitoSox assay showed a decrease in mitochondrial superoxide in FTH1-deficient cells (FTH1-KD: MitoSOX Red MFI: 251,8 vs. SCR: MitoSOX Red MFI: 1002,3) (Figure 2B). A similar, asymmetric behavior was observed by flow cytometry analysis, which revealed a reduction in TMRM fluorescence, indicative of mitochondrial membrane depolarization, in FTH1-KD hESCs (MFI: 7722) compared to SCR (MFI: 11086,7) (Figure 2C). Altogether, these results led us to conclude that hESCs activate a powerful and effective antioxidant mechanism and are thus capable of protect themselves, at least to some extent, from shFTH1-mediated iron toxicity. 

### 3.3. Nrf2 Signaling Pathway Drives the Antioxidant Response in hESCs

**Figure 3:**
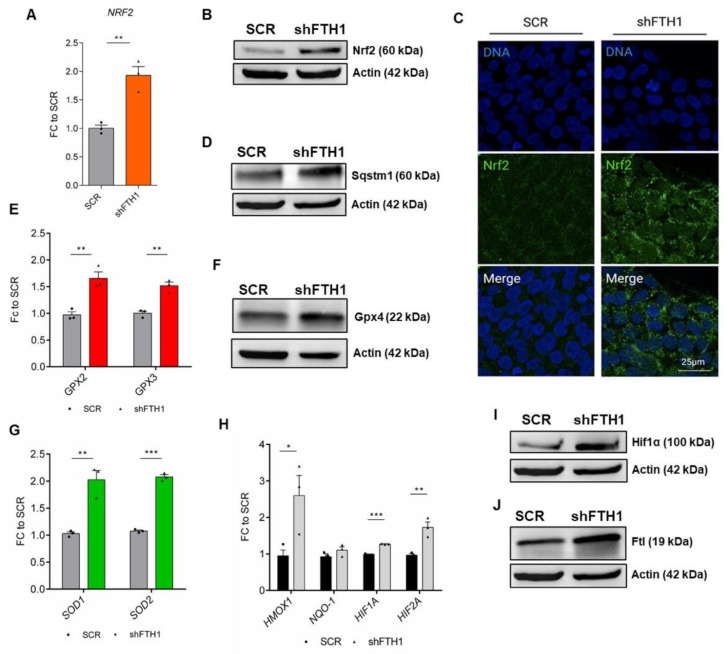
The Nrf2 pathway is activated in FTH1-KD hESCs. (**A**) *NRF2* expression was measured in undifferentiated SCR and FTH1-KD hESCs via qRT-PCR, showing a significant up-regulation in presence of FTH1 silencing; (**B**) Nrf2 protein level was assessed by immunoblotting (IB), indicating an increased expression of FTH1-KD hESCs; (**C**) Immunofluorescence of Nrf2 (green) and nuclei (blue) in SCR control hESCs and FTH1 silenced cells (scale bar, 25 µm); (**D**) Immunoblot analysis for p62/Sqstm1 protein in SCR and FTH1-KD cells showing an upregulation of its expression in silenced cells; (**E**) qRT-PCR analysis of glutathione peroxidases 2 and 3 (*GPX2*, *GPX3*) antioxidant genes in SCR control and FTH1-KD hESCs; (**F**) Western blot analysis for Gpx4 protein, confirming increased expression of FTH1-KD hESCs; (**G**) qRT-PCR analysis of *SOD1* and *SOD2* expression in SCR and shFTH1 hESCs showing an upregulation of these genes in modulated hESCs; (**H**) mRNA levels of Nrf2 target genes (*HMOX1*, *NQO-1*, HIF1α, HIF2α) were measured in SCR and FTH1-KD hESCs via qRT-PCR; (**I, J**) Immunoblot analysis of HIF1α (**I**) and ferritin light chain (Ftl) (**J**) showed upregulation of both proteins in FTH1-silenced cells with respect to SCR cells. In all Western blots presented in this figure, actin levels were evaluated to confirm equal loading control. qRT-PCR data were normalized to values in SCR cells and are represented as mean ± SEM (*n* = 3); significance is calculated by *t*-test: * *p* ≤ 0.05; ** *p* ≤ 0.01, *** *p* ≤ 0.001. Small dots refer to SCR replicates, while triangles refer to shFTH1 triplicates.

Nuclear factor erythroid 2-related factor 2 (Nrf2) is a transcription factor ubiquitously expressed in most eukaryotic cells. Nrf2-Keap1 (Kelch-like ECH-associated protein 1) signaling pathway was shown to play a central role in protecting cells against oxidative stress [37,38]. Under basal conditions, Nrf2 is bound to its negative regulator Keap1, which directs it to proteasomal degradation. In the presence of high levels of intracellular ROS, Keap1 is oxidized and releases Nrf2, which is free to translocate into the nucleus, where it binds to antioxidant response elements (AREs) and induces the expression of antioxidant target genes, such as *HMOX1*, *NQO1*, *GST*, and *GPX* [39]. Besides its role as a fine regulator of redox and metabolic homeostasis, Nrf2 also acts as a pluripotency master gene: Nrf2 activation in hESCs was shown to enhance *NANOG* transcriptional activity by delaying Nanog protein degradation through POMP-mediated proteasome ubiquitination [40,41]. In this study, we found that FTH1-KD hESCs express higher levels of Nrf2 transcript and protein, assessed via qRT-PCR (Figure 3A), immunoblot (Figure 3B and Supplementary Figure S4A), and immunofluorescence (Figure 3C and Supplementary Figure S4F), respectively. Similarly, we could also observe an increased expression of p62/Sqstm1 (Figure 3D and Supplementary Figure S4B) which, in the non-canonical Nrf2-Keap1 pathway, is known to bind Keap1, allowing Nrf2 to migrate into the nucleus [42]. Moreover, the p62 protein yields a feedback loop that amplifies the Nrf2 system [43]. In addition to increased expression of Nrf2 in FTH1-silenced hESCs, we observed the overexpression of major antioxidant enzymes, such as glutathione peroxidases *GPX2*, *GPX3* (Figure 3E), Gpx4 (Figure 3F and Supplementary Figure S4C), and superoxide dismutases (*SOD*) *1* and *2* (Figure 3G). Additionally, several Nrf2-regulated genes (*HMOX1*, *NQO-1*, HIF1α, HIF2α) (Figure 3H) and proteins (HIF1α, Ftl) (Figure 3I,J respectively and Supplementary Figure S4D,E) were found to be highly expressed in FTH1-KD hESCs. Altogether, these findings suggest a crosstalk between *FTH1* silencing and overactivation of the Nrf2 signaling pathway and its cognate effectors in hESCs. 

### 3.4. Effects of FTH1 Knock-Down on Apoptosis 

**Figure 4:**
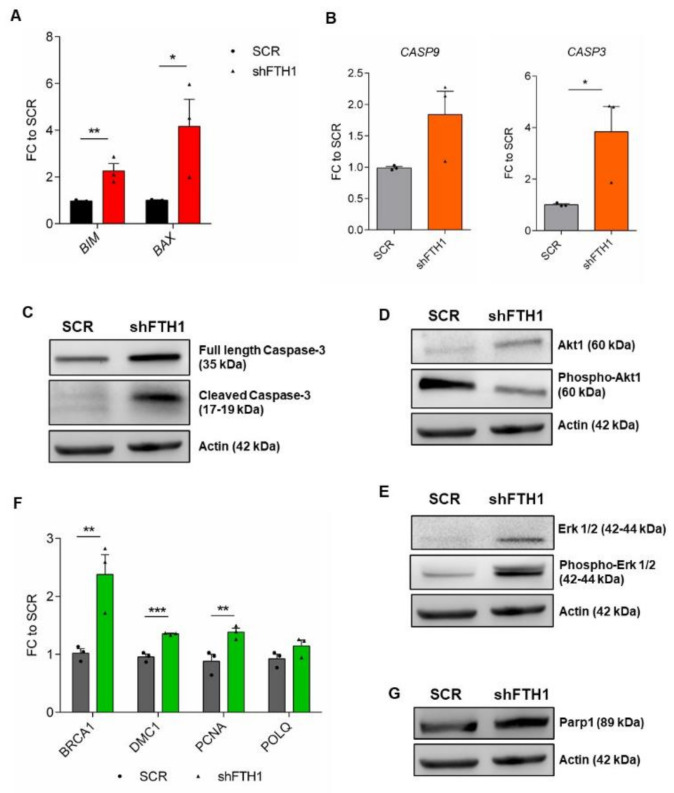
*FTH1* silencing leads to expression of apoptosis and DNA damage markers in hESCs. (**A**,**B**) qRT-PCR for apoptosis-associated genes *BIM* and *BAX* (**A**) and *CASP3* and *CASP9* (**B**) showing increased expression levels of apoptotic marker in FTH1-KD hESCs compared to SCR control; (**C**) Activation of apoptotic signaling in FTH1-KD hESCs is demonstrated also by immunoblot analysis for full length and cleaved form of caspase-3; (**D**) Western blot analysis of Akt1 and phosphorylated Akt1 (Ser473, pAkt) protein expression; (**E**) Immunoblot for total Erk1/2 and phosphorylated Erk1/2 (Thr202/Tyr204, pErk1/2) in SCR control and FTH1-KD hESCs; (**F**) qRT-PCR analysis for DNA damage related genes (*BRCA1*, *DMC1*, *PCNA*, *POLQ*) in control and silenced hESCs; (**G**) Western blot analysis for Parp1 protein expression in SCR and FTH1-silenced cells. For all Western blots shown, actin levels were evaluated as loading control. Results of qRT-PCR were normalized to measurements in SCR cells (*n* = 3); * *p* ≤ 0.05; ** *p* ≤ 0.01, *t*-test, *** *p* ≤ 0.001. Small dots refer to SCR replicates, while triangles refer to shFTH1 triplicates.

Labile iron accumulation is a well-known cell damage effector and pro-apoptotic factor [44,45,46]; intracellular iron overload by ferric ammonium citrate (FAC) treatment leads to ferroptosis, an iron-regulated cell death [47]. To check the effects of FTH1-silencing on apoptosis, expression analysis of intrinsic pro-apoptotic genes such as *BAX* and *BIM*, as well as *CASP9* and *CASP3* was performed, revealing a significant upregulation in FTH1-KD hESCs compared to SCR control (Figure 4A,B). Similarly, immunoblot analysis of cleaved Caspase3 protein confirmed its overexpression in FTH1-silenced hESCs (Figure 4C, Supplementary Figure S5A,E). Further, flow cytometry-based analysis of apoptosis showed a significantly higher percentage of late apoptotic cells in FTH1-KD cells compared to SCR (Supplementary Figure S7). It is by now generally accepted that Akt and Erk1/2 pathways are both linked to apoptosis, but with opposite effects: Erk promotes apoptosis, both intrinsically and extrinsically and its activity can be driven by the presence of ROS [48]. By contrast, Akt is linked to cell survival, therefore its activity mediates the suppression of apoptosis [49]. Based on these observations, we speculated whether Erk and Akt could be involved in our FTH1-KD hESCs system; therefore, we measured their expression levels by immunoblot analysis and found that the active, phosphorylated Akt protein (pAkt) expression was reduced (Figure 4D, Supplementary Figure S5B,F), while the expression of pErk1/2 resulted in a significant increase (Figure 4E, Supplementary Figure S5C,G) in FTH1-silenced hESCs. Collectively, these findings clearly suggest that an active cell death program occurs in hESCs when ferritin is stably downregulated. In addition, based on the evidence that high ROS levels and *FTH1* modulation also associate with DNA damage [50,51,52,53], we measured the expression of some DNA damage-associated genes. In line with previous studies, here we show that *BRCA1*, *DMC1*, *PCNA*, and *POLQ* are indeed significantly upregulated in FTH1-KD hESCs (Figure 4F). Similarly, we could also observe an increased expression of Parp1 protein, a substrate of activated caspase 3, in FTH1-silenced hESCs (Figure 4G, Supplementary Figure S5D,H). Overall, these results provide strong evidence that *FTH1* silencing induces apoptosis in hESCs and triggers the activation of DNA-damage response programs, which are efficiently counteracted by the survival mechanisms described above. 

### 3.5. Effects of FTH1 Repression on Cellular Metabolism

**Figure 5:**
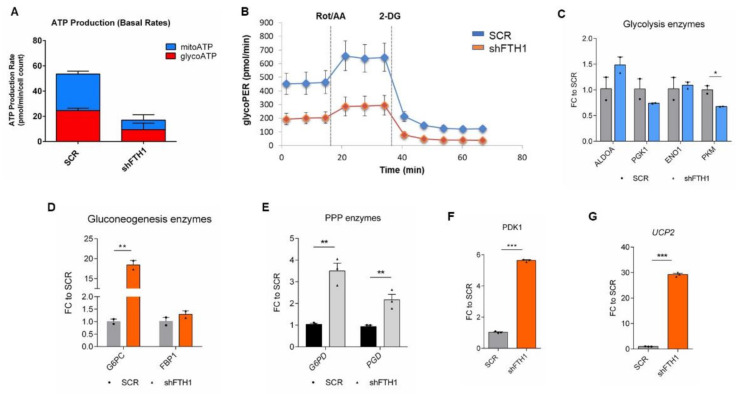
Metabolic rearrangement in FTH1-KD hESCs. (**A**) Quantification of total cellular ATP production (from glycolysis and mitochondrial oxidative phosphorylation) rate using a Seahorse XF analyzer. ATP production was calculated by measuring the oxygen consumption and acidification rate. Each sample (SCR and shFTH1-hESCs) was measured in at least three biological replicates. Seahorse analysis shows a lower ATP production (from both sources) in FTH1-KD cells compared to SCR control; (**B**) Glycolytic rate measurements in SCR and FTH1-silenced cells calculated by subtracting extracellular acidification rate (ECAR). As shown, FTH1-KD hESCs do not undergo metabolic switch. Since mitochondrial acidification is blocked, the approach measures glycolytic-ECAR only correlating to extracellular lactate accumulation. Glycolytic rate was reduced in FTH1-silenced hESCs; (**C**) qRT-PCR analysis for glycolytic enzymes (*ALDOA2*, *PGK1*, *ENO1*, and *PKM*) mRNA expression; (**D**) Expression of *G6PC* and *FBP1* gluconeogenesis genes in SCR and shFTH1-hESCs measured by qRT-PCR analysis; (**E**) Pentose phosphate pathway (PPP) genes *G6PD* and *PGD* were significantly up-regulated shFTH1 cells with respect to SCR control cells; (**F**,**G**) The *PDK1* gene, responsible for inactivation of pyruvate dehydrogenase (**F**) and the gene *UCP2* (**G**), which uncouples OXPHOS from ATP synthesis, both resulted in significantly up-regulated in FTH1-KD cells compared to SCR cells. For all qRT-PCR graphs, results were normalized to measurements in SCR cells (*n* = 3); * *p* ≤ 0.05; ** *p* ≤ 0.01, *** *p* ≤ 0.001, *t*-test compared to SCR. Small dots refer to SCR replicates, while triangles refer to shFTH1 triplicates.

Under oxidative stress conditions, a metabolic shift from OXPHOS to glycolysis was reported to be occurring [54], indicating a clear and intimate correlation between oxidative stress and metabolic changes. It is also well known that hPSCs mainly rely on glycolysis while OXPHOS takes place as soon as these cells enter differentiation [33,55]. Using the Seahorse analyzer, we first quantified the fraction of ATP generated by glycolysis and by OXPHOS, demonstrating an overall reduction in total ATP production from both metabolic sources in FTH1-silenced hESCs compared to SCR control. Therefore, we further sought to speculate on the total glycolytic activity in both cell lines. A lower basal and compensatory glycolytic rate was detected in FTH1-silenced hESCs, suggesting that mitochondrial respiration is somehow inhibited in these cells, which retain the capability to manage energy demand despite the downregulation of the ferritin gene (Figure 5B and Supplementary Figure S6B). For a more comprehensive metabolic investigation, we analyzed the expression levels of glycolysis-specific genes such as *ALDOA*, *PGK1*, *ENO1*, and *PKM*, along with gluconeogenesis-related genes *G6PC1* and *FBP1.* In line with the results obtained by the glycolytic rate analysis, we could not observe significant changes in the expression of glycolytic genes between SCR and FTH1-silenced hESCs, with the exception of the PKM (pyruvate kinase M1/2) gene, encoding an ATP-generating enzyme, which results in significantly downregulated silenced cells (Figure 5C), strengthening the result obtained with the Seahorse analyzer quantification of total ATP production. On the other hand, the expression of *G6PC1* (Glucose-6-Phosphatase Catalytic Subunit 1) resulted in significantly higher FTH1-silenced cells (Figure 5D). *G6PC1* encodes one of the key enzymes responsible for glucose production from glucose-6-phosphate, and therefore it is critically involved in glucose homeostasis. This finding perfectly matches with the fact that high glucose levels are required for the proper functionality of detox systems, such as the pentose phosphate (PPP), glucuronidation, and glutathione biosynthesis pathways, all of which are finely tuned by Nrf2 [56,57]. In support of this, we found that *G6PD* (glucose-6-phosphate dehydrogenase) and *PGD* (6-Phosphogluconate dehydrogenase), two key enzymes of the PPP pathway, were significantly increased in FTH1-silenced cells (Figure 5E), strengthening the concept that the PPP may represent a crucial metabolic target through which the Nrf2 signaling potentiates the antioxidant response in hPSCs. The expression of *PDK1* (pyruvate dehydrogenase kinase 1) was also significantly enhanced in FTH1-silenced hESCs (Figure 5F); its expression is positively regulated by HIF1α, previously shown to participate in ROS-induced metabolic reprogramming, where *PDK1* prevents the pyruvate from entering the TCA cycle (tricarboxylic acid cycle or Krebs cycle) [58]. We finally tested the expression level of *UCP2* (Uncoupling protein-2), a protein of the inner mitochondrial membrane, involved in uncoupling OXPHOS from ATP synthesis. Analogously to *PDK1*, *UCP2* expression was significantly higher in FTH1-silenced hESCs (Figure 5G). As *PDK1*, *UCP2* blocks pyruvate entry into the Krebs cycle and shunts it towards the PPP pathway [19,59,60]. Altogether, our findings are indicative of a metabolic rewiring occurring in response to iron-mediated oxidative stress in hESCs.

## 4. Discussion

Pluripotent stem cells (PSCs), including ESCs and iPSCs, possess unique and extraordinary self-renewal capabilities, while maintaining their pluripotency [61]. In addition to these properties, PSCs also have the ability to give rise to almost any cell type, both in vivo and in vitro. On the basis of these key features, PSCs technology holds enormous potential in many fields of biomedical research, including drug screening, development and repurposing [62,63,64,65], disease modelling [66,67,68,69,70], cell therapy [71], aging [72], and regenerative medicine [73]. Although significant progress has been made, many hurdles still need to be overcome to push PSCs technology towards the road to clinical practice. Apart from immunogenicity and genomic instability, undoubtedly representing the two major challenges, the successful differentiation and functional maturation of PSC derivatives must faithfully recapitulate all aspects of somatic cells and tissues, including the metabolic function. For instance, little is known about the role of ferritin heavy chain (FTH1), the major ferroxidase protein, in human ESCs homeostasis. Ferritin is composed of 24 heavy and light chains, whose core is able to sequester up to 4500 iron atoms [74]. Ferritin heavy chain, in particular, is responsible for the oxidation of ferrous iron [Fe(II)] to less reactive ferric iron [Fe(III)] [75]. Even though there is a wide literature suggesting that FTH1 is a major player in protecting cells against oxidative stress [76], so far, its role in embryonic stem cells has not been fully elucidated. As mentioned earlier in this work, ROS play an important role as second messengers in embryo development [77,78], and their effects, beneficial or deleterious, depend on the embryo developmental stage. Compared to most terminally differentiated cells, ESCs are extremely resistant to hypoxic stress as they live and greatly proliferate at low oxygen concentrations [79,80], suggesting that they are equipped with a highly efficient antioxidant system [5,10]. Because of its strong ferroxidase activity, FTH1 is supposed to be one of the major players in this system, maintaining iron in a safe and bioavailable form. To gain insights on the effects of FTH1-mediated control of ROS-induced cellular stress, here we stably silenced the FTH1 gene in H9 human embryonic stem cell line using the shRNA strategy. Intracellular ROS production, measured in shFTH1-hESC by flow cytometry together with mitochondrial superoxide species, revealed that silenced cells have decreased levels of ROS accumulation. Similarly, mitochondrial membrane potential detected by TMRM assay, showed that FTH1 silencing produces a reduction in mitochondrial membrane depolarization in shFTH1-hESCs compared to SCR control. By monitoring self-renewal and pluripotency, interesting findings emerged: (i) morphologically, shFTH1 hESCs were more flattened and had less AP+ cells compared to SCR control cells; (ii) the core pluripotency genes *OCT4, NANOG*, and *SOX2* were significantly overexpressed in FTH1-silenced cells; (iii) immunoblot analysis of Nanog protein revealed an increased expression in silenced cells. As pointed out elsewhere in the text, NANOG expression is under the control of the nuclear factor erythroid 2-related factor 2 (Nrf2), a stress-activated transcription factor involved in the cellular response to multiple injuries, among which oxidation is the most prominent. In a recent review, Dai and Coll. [81] summarize Nrf2-Keap1 signaling in the context of stem cell state and function, suggesting that it can serve as a master regulator of stem cell redox and metabolic homeostasis. Nrf2, in fact, can efficiently modulate ESCs and iPSCs pluripotency status by finely tuning NANOG and OCT4 expression through direct binding to upstream regions of these genes and delaying their proteasome-mediated ubiquitination [41]. Our data showed clear evidence of the increased Nrf2 expression in hESCs, both at mRNA and protein level, upon FTH1 silencing. Overexpression of Nrf2 is therefore responsible, in our model, for the upregulation of NANOG, as well as for the activation of the Gpx4 (glutathione peroxidase 4) ROS scavenger protein. A correlation between activation of Nrf2 and induced expression of FTH1 has been previously reported in mouse embryonic fibroblasts (MEFs) exposed to beta-naphthoflavone and chemo preventive dithiolethiones, providing a mechanistic link between regulation of the iron storage protein ferritin and the cancer chemo preventive response [82]. Ferritin is also known to function as a powerful anti-apoptotic protein [83]. Accordingly, our results show that FTH1-silencing triggers the activation of apoptosis-associated genes *BAX*, *BIM*, *CASP3*, and *CASP9*; we further deepened our analysis by studying the AKT and ERK1/2 pathways, which undergo significant perturbations during apoptosis. Previous studies have reported conflicting results, showing that MAPK/ERK and PI3/AKT signaling pathways can be either activated [84] or decreased [85] in mESCs and hESCs upon ROS exposure. Our data clearly indicate that ferritin silencing produces a reduction in the levels of the active, phosphorylated form of Akt, while ERK1/2 signaling is activated. With respect to cell metabolism, most of the studies have focused on glycolytic metabolism and on its role in maintaining pluripotency, as well as the importance of a metabolic shift required for somatic cells in order to enter pluripotency during reprogramming. In this study we went beyond previous reports, speculating whether modulation of FTH1 by repression may impact on hESCs homeostasis and metabolic properties. Interestingly, extensive analysis showed that FTH1-silenced hESCs undergo significant metabolic reorganization. More specifically, we observed that FTH1-KD hESCs cells respond to the loss of ferritin heavy chain by activating a strong antioxidant response that entails the expression of major ROS scavengers such as SODs, CAT, and GPXs. Moreover, FTH1-silenced hESCs had an increased expression of the *G6PD* gene, which is one of the most important sources of NADPH, essential for maintaining redox homeostasis [86]. Together with *G6PD*, *PDK*1 and *UCP2* were upregulated as well in silenced cells, suggesting a rewiring of metabolites from glycolysis to PPP and corroborated by a reduced glycolytic flux in shFTH1-hESCs. Consistent with the idea that PPP flux leads to an elevated reduction of NADP+ to NADPH [87,88], we suggest that an increased PPP flux is favored in hESCs with repressed FTH1 expression to drive the ROS clearance.

## 5. Conclusions

**Figure 6:**
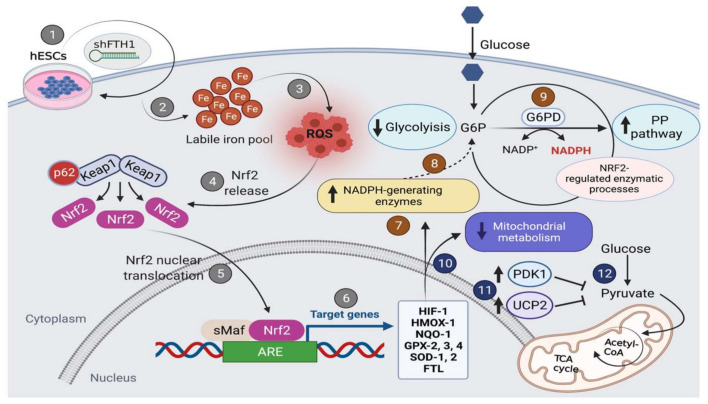
Schematic diagram illustrating oxidative stress response mediated by FTH1 downregulation in hESCs. FTH1 (1) is the master scavenger molecule for intracellular iron homeostasis and its inactivation leads to imbalanced redox homeostasis due to intracellular iron accumulation (2) and ROS production (3). Surprisingly, in hESCs, a reduced expression of FTH1 expression is not associated with oxidative stress and ROS production. This phenomenon can be explained by three crucial findings: first, FTH1-silenced hESCs showed an enhanced transcriptional activity of Nrf2 (4–5) on key antioxidant target genes such as *HIF-1*, *HMOX-1*, *NQO-1*, *GPX2/3/4*, *SOD1/2*, *FTL* (6); second, we observed the activation of an alternative metabolic program from glycolysis (7–8) to pentose phosphate pathway (PPP) where the activity of the G6PD enzyme ensures the conversion of NADP+ to NADPH (9) involved in ROS neutralization; third, the mitochondrial metabolism was found to be inactive (10) in FTH1-silenced hESCs, as demonstrated by the upregulation of *PDK1* and *UCP2* genes (11), specifically involved in preventing pyruvate from entering the tricarboxylic acid cycle (TCA) (12).

To our knowledge, this is the first study shedding light on the role of ferritin modulation by repression in human ESCs. In agreement with our work, previous studies have reported that ESCs are very resistant to oxidative stress. Interestingly, here we provide evidence of a potential interplay between the Nrf2 axis and oxidative pentose phosphate pathway (PPP) as the molecular mechanism underlying the antioxidant response in hESCs under shFTH1-induced stress conditions (Figure 6). Future investigations are necessary to validate the conclusions drawn from this study.

## Data Availability

All the data presented in this study are available from the corresponding author upon reasonable request.

## Supplementary Materials

The following are available online at https://www.mdpi.com/article/10.3390/cells10092431/s1, Figure S1: Assessment of FTH1 silencing efficiency, Figure S2: SCR and shFTH1 hESCs pluripotency characterization, Figure S3: Iron level quantification and flow cytometric assessment of intracellular ROS levels and mitochondrial membrane potential (ΔΨM), Figure S4: Study of Nrf2 signaling activation upon FTH1 silencing, Figure S5: Evaluation of FTH1 silencing effect on apoptosis and DNA damage, Figure S6: Analysis of metabolic changes occurring in FTH1 hESCs. Table S1: List of primers used for qRT-PCR experiments.
