# Supplementary material for: Uncovering the Metabolic and Stress Responses of Human Embryonic Stem Cells to FTH1 Gene Silencing
Source: Cells. 2021 Sep 15;10(9):2431. doi: 10.3390/cells10092431 (PMC8469604; doi:10.3390/cells10092431)
Supplement: Supplementary file 1 [file cells-10-02431-s001.zip › cells-1366337-supplementary.pdf]

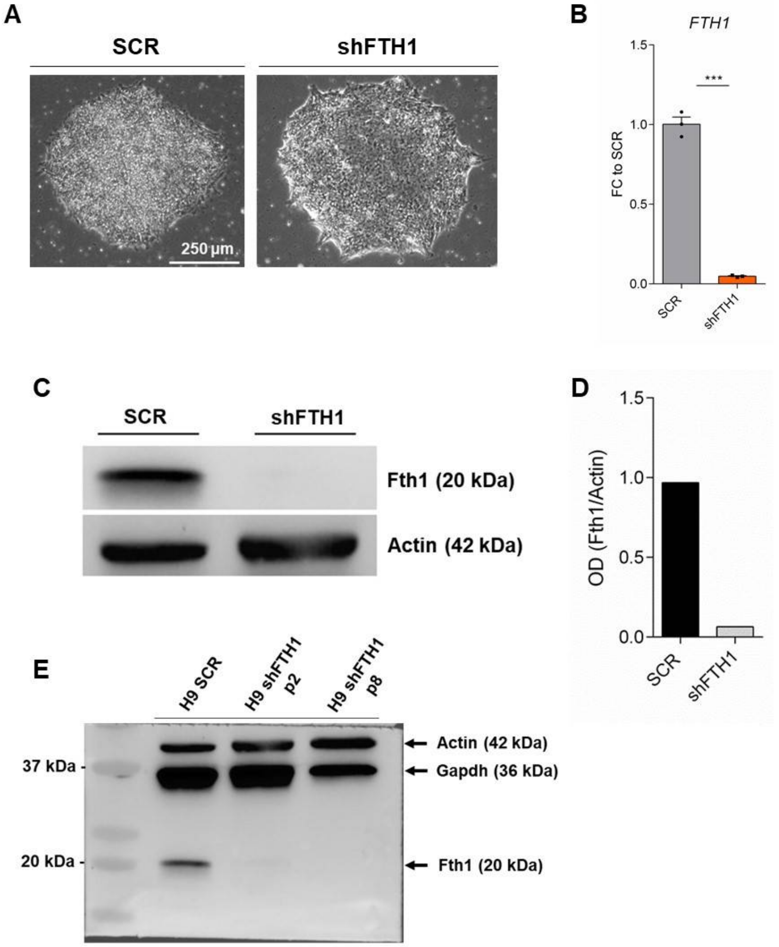

Figure S1: Assessment of FTH1 silencing efficiency. (A) Brightfield images of hESC colonies; **(B)** qRT-PCR analysis of *FTH1* expression transcript reveals reduced levels in ESCs after infection with shRNA lentiviral targeting *FTH1* (sh*FTH1*). $^{***}P<0.001$ *vs.* scramble (SCR); *t*-test; **(C)** Western blotting using an antibody directed against Fth1 shows reduced expression of the protein (20 kDa) in total lysates of shFTH1-infected ESCs. Actin is shown as a loading control; **(D)** Densitometric readings reveal an almost 80% FTH1 knock-down efficiency in shFTH1-infected cells compared to their SCR control. Values are expressed as the integrals (area x mean density) of each band normalized to Actin and relative to SCR; **(E)** Uncropped original image of Western Blot analysis of Fth1 knockdown (shown in Fig. S1C).

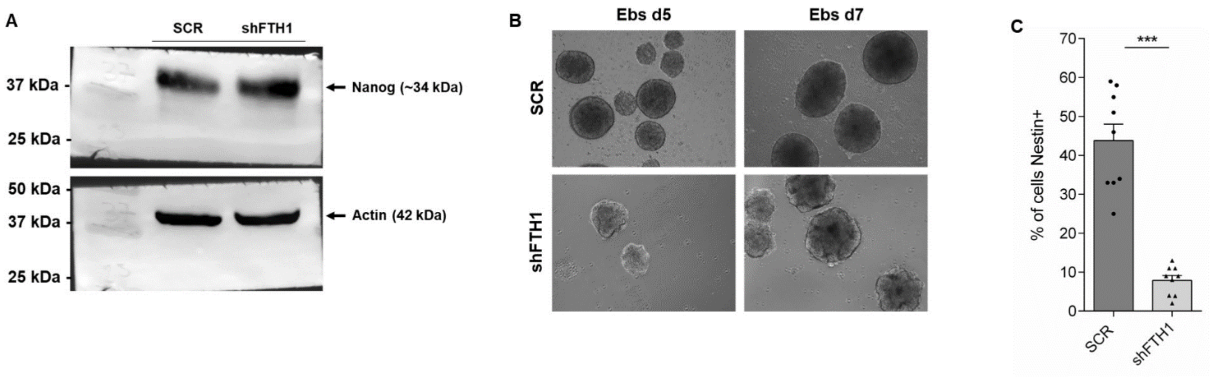

Figure S2: SCR and shFTH1 hESCs pluripotency characterization. (A) Uncropped original image of Western blot analysis for Figure 1C; **(B)** Brightfield images of floating embryoid bodies (EBs), obtained from SCR and shFTH1-hESCs, on day 5 and day 7 of differentiation; **(C)** Quantification of the percentage of Nestin-positive cells in SCR and shFTH1 EBs on day 28 of differentiation using ImageJ (immunofluorescence shown in Fig. 1F). Data are represented as mean ± SEM (n=9); significance is calculated by *t*-test: ***P≤0.001.

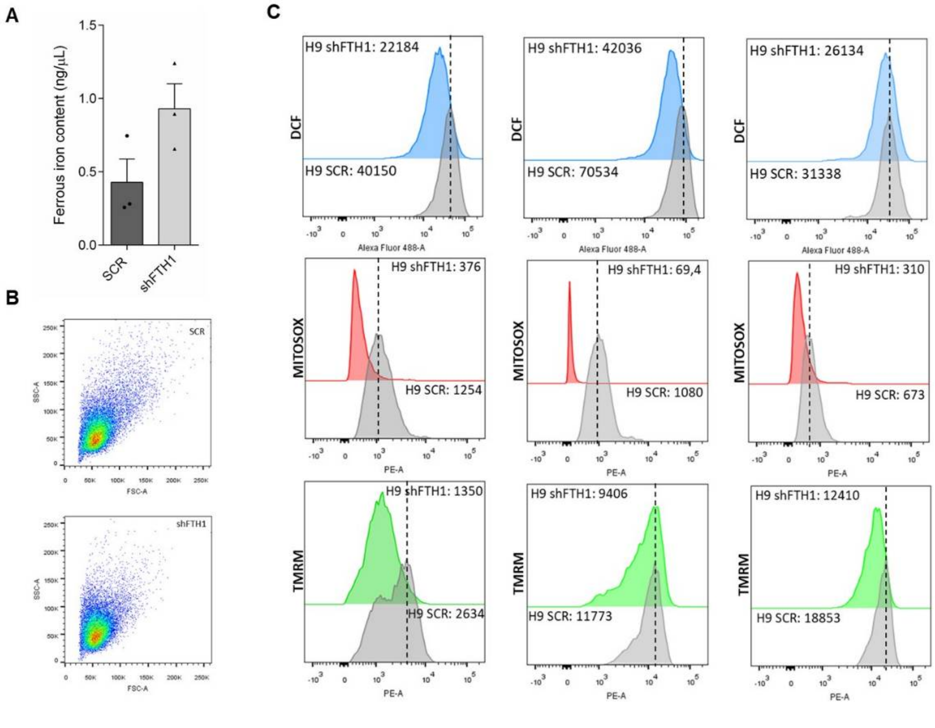

Figure S3: Iron levels quantification and flow cytometric assessment of intracellular ROS levels and mitochondrial membrane potential (ΔΨM). (A) Ferrous iron content quantification (colorimetric detection). Data are represented as mean ± SEM (n=3); **(B)** Forward and side scatter of SCR and shFTH1 hESCs measured by flow cytometry (density plots); **(C)** Quantification of total intracytoplasmic ROS and specific mitochondrial superoxide species (using DCF and MitoSox, respectively) and analysis of mitochondrial membrane potential (ΔΨM) (using TMRM) in SCR and shFTH1 hESCs shown in Figure 2.

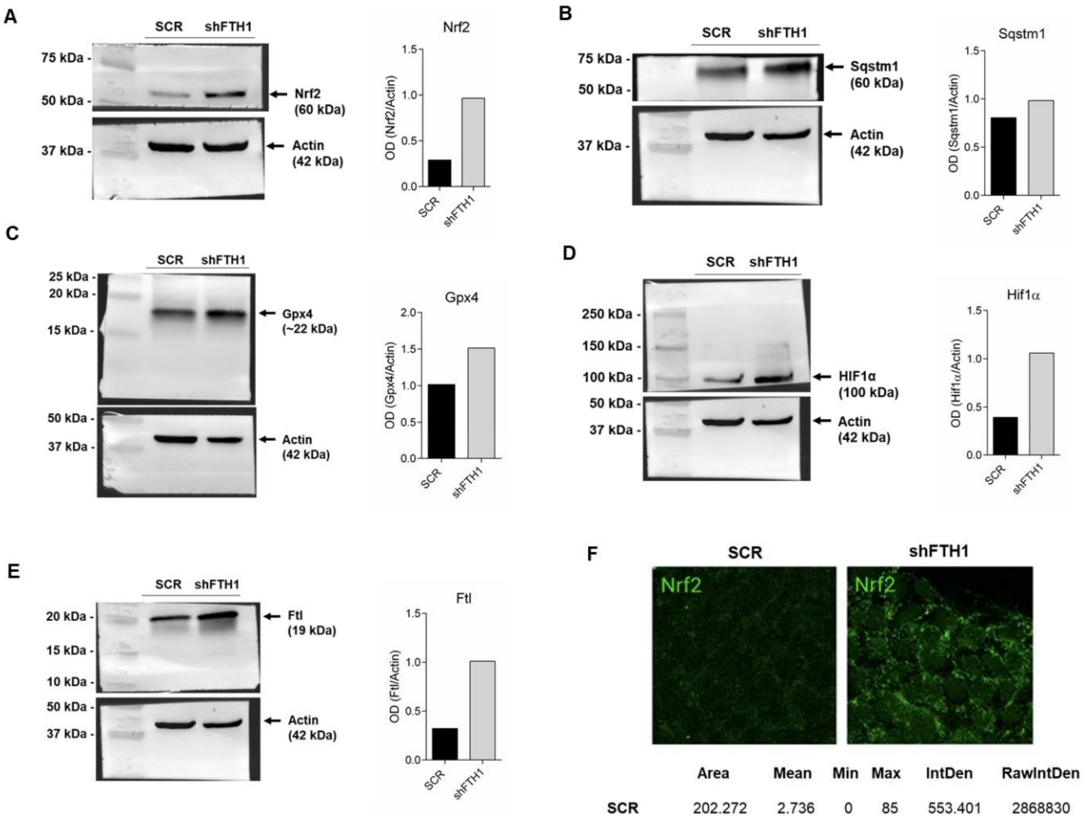

	Area	Mean	Min	Max	IntDen	RawIntDen
SCR	202.272	2.736	0	85	553.401	2868830
shFTH1	202.272	11.387	0	85	2303.300	11940308

Figure S4: Study of Nrf2 signalling activation upon FTH1 silencing. (A-E) Uncropped original images of Western blot analyses for Figure 3 (**A:** Nrf2, **B:** Sqstm1, **C:** Gpx4, **D:** Hif1α, **E:** Ftl) and relative densitometric readings (bar plots); **(F)** Quantification of fluorescence intensity signal of Nrf2 using Fiji software (immunofluorescence shown in Figure 3C).

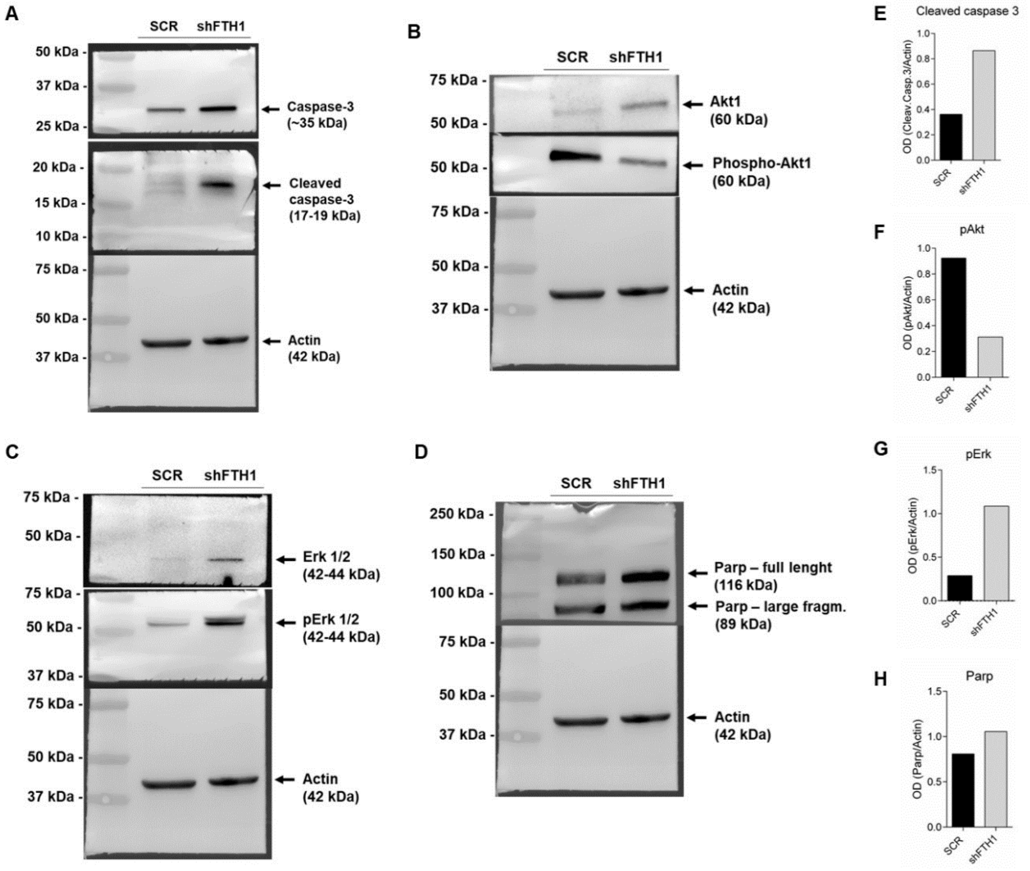

Figure S5: Evaluation of FTH1 silencing effect on apoptosis and DNA damage. (A-D) Uncropped original images of Western blot analyses for Figure 4 (**A:** Caspase-3, **B:** Akt1, **C:** Erk 1/2, **D:** Parp); (**E**-**H**) Densitometric readings relative to Western blots in Figure 4 (bar plots).

(A) XF Real-Time ATP Rate Assay

	mitoATP Production Rate		glycoATP Production Rate		ATP Production Rate		XF ATP Rate Index		% Glycolysis		% Oxidative Phosphorylation	
Groups	Value	Std Dev	Value	Std Dev	Value	Std Dev	Value	Std Dev	Value	Std Dev	Value	Std Dev
SCR	029	002	025	002	053	004	001	000	046	001	054	001
shFTH1	008	004	009	005	017	009	001	000	055	004	045	004

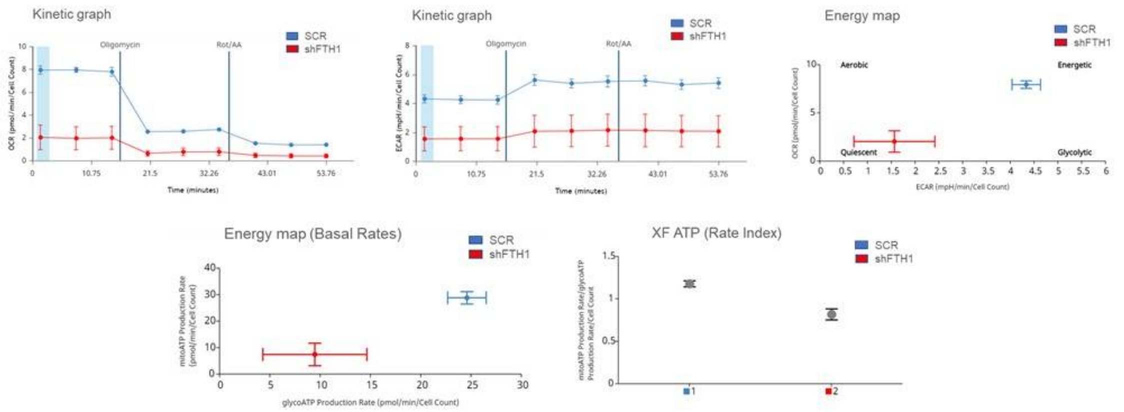

(B) Glycolytic Rate Assay

	Basal Glycolysis		Basal Proton Efflux Rate		% PER from Glycolysis		Compensatory Glycolysis		mitoOCR/glycoPER (Basal)		Post 2-DG Acidification (glycoPER)	
Groups	Value	Std Dev	Value	Std Dev	Value	Std Dev	Value	Std Dev	Value	Std Dev	Value	Std Dev
SCR	465	084	509	092	091	001	658	110	000	000	122	019
shFTH1	204	041	224	049	092	002	295	073	000	000	039	015

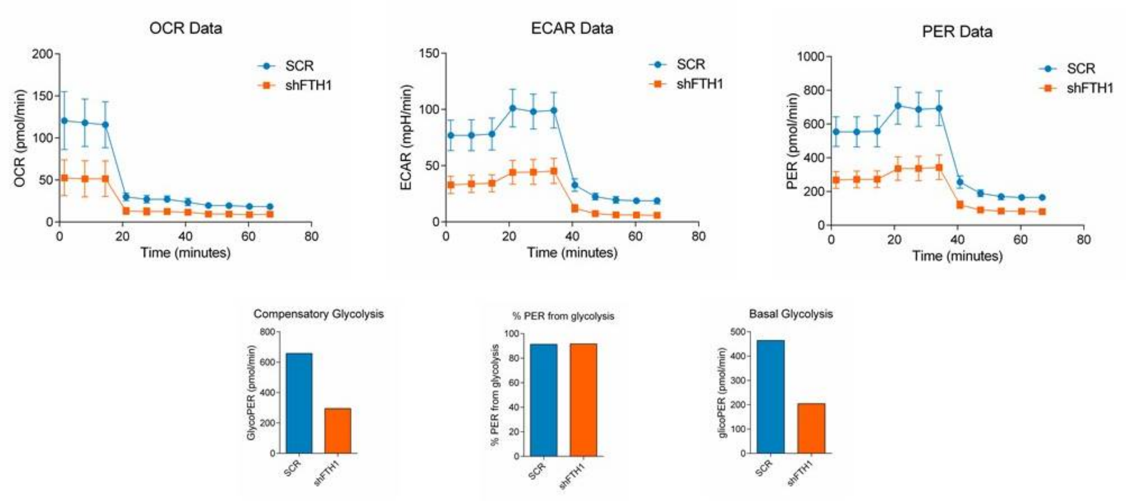

Figure S6: Analysis of metabolic changes occurring in FTH1 hESCs. (A) MitoATP and glycoATP production rates evaluated in SCR and shFTH1 hESCs using XF-Real Time ATP Rate Assay. “Kinetic graphs” indicate the oxygen consumption rate (OCR) deriving from the mitochondrial respiration and the extracellular acidification (ECAR), which allows the calculation of total Proton Efflux Rate (PER), before and after the injection of oligomycin and Rotenone + Antimycin A (Rot/AA). “Energy map” for OCR and ECAR shows four relative bioenergetic phenotypes that SCR and shFTH1 hESCs can display. Quiescent indicates that cells don’t use mitochondrial respiration or glycolysis. Energetic is for cells that utilize both metabolic processes. “Energy map (Basal Rates)” indicates mitoATP Production Rate and glycoATP Production Rate. “XF ATP (Rate index)” explains the ratio between the mitoATP Production Rate and the glycoATP Production Rate at a specific time point. Analysis of ATP production rate in SCR and shFTH1 hESCs is shown in Figure 5, **(B)** Glycolytic Rate Assay for the measurement of glycolysis in SCR and shFTH1 hESCs. Oxygen consumption rate (OCR) and extracellular acidification rate (ECAR) show the production of energy deriving from glycolysis and oxidative phosphorylation. Proton Efflux Rate (PER) is calculated after the inhibition of the mitochondrial respiration with Rot/AA. PER can be subtracted from the total proton efflux, determining the glycolytic proton efflux rate (glycoPER). “Compensatory glycolysis” shows the glycolytic rate after the injection of Rot/AA. Analysis of glycolytic rate in SCR and shFTH1 hESC is shown in Figure 5.

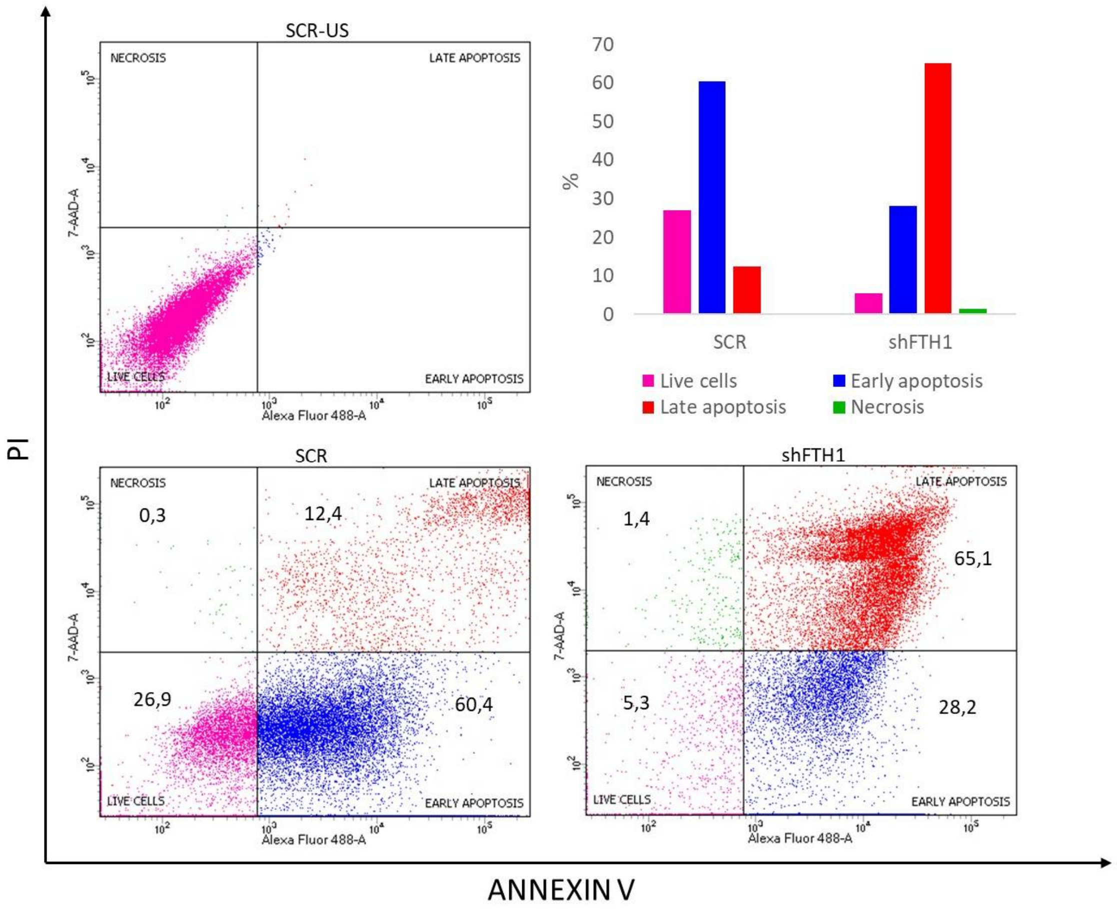

Figure S7: Quantification of the percentage of apoptotic cells in SCR and shFTH1 hESCs. The number of early apoptotic, necrotic and late apoptotic cells (Annexin V, PI and Annexin V+PI labeled cells, respectively) was quantified using flow cytometry. The results were analyzed with FlowJo software (Tree Star, Inc.).
